# Alcool, Alcoolisme, Aloolisation

**Published:** 1995

**Authors:** Philip J. Cook, Ole-Jørgen Skog

**Affiliations:** Philip J. Cook, Ph.D., is Sanford Professor of Public Policy Studies, Terry Sanford Institute of Public Policy, Duke University, Durham, North Carolina. Ole-Jørgen Skog is director of research for SIFA, the State alcohol monopoly in Oslo, Norway

**Keywords:** AOD consumption, heavy AOD use, AOD-related (AODR) mortality

**Figure:**
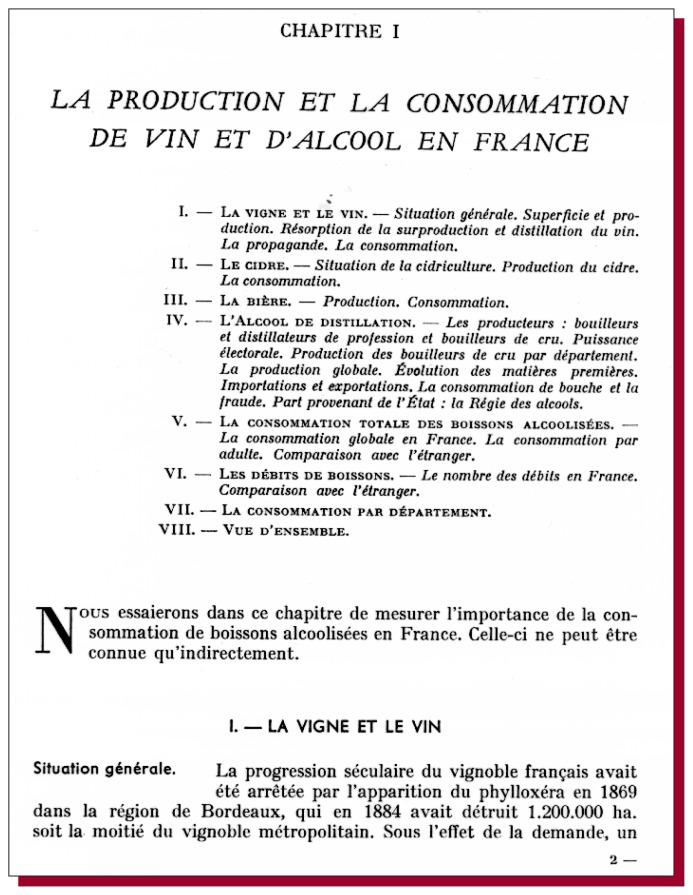
Ledermann, S. *Alcool, Alcoolisme, Alcoolisation*. Vol. 1. Données Scientifique de Caractère Physiologique, Économique et Social. (Institute Nationale d’Etudes Demographique, Travaux et Documents, Cah. No. 29.) Paris: Presses Universitaires de France, 1956.

In this treatise published in 1956, the French demographer Ledermann proposed his “single distribution theory” of alcohol consumption. In subsequent decades, this still controversial theory has stimulated research that has contributed to a fundamental reconsideration of heavy drinking and alcoholism. Public health advocates have used Ledermann’s theory to justify higher taxes and other limits on the availability of alcohol to the public.

## The Single Distribution Theory

Ledermann’s earlier work included a series of studies on the relationship between mortality patterns and the general level of alcohol consumption in a population, both over time and across France. He demonstrated that regions in which average consumption was high tended to have relatively high mortality from liver cirrhosis and other alcohol-related causes, suggesting that the prevalence of heavy drinking—and drinking-related health consequences—in a population is closely related to the general level of drinking in that population.

In *Alcool, Alcoolisme, Alcoolisation*, Ledermann offered several general concepts, two of which are described below, that fit his epidemiological findings but went much farther:

1. A graph depicting the percentage of drinkers at each level of alcohol consumption (from one drink per year to thousands of drinks per year) at a given point in time would have the characteristic shape of the lognormal curve. This asymetric curve would show a single peak (indicating the highest concentration of people). The “tail” extending to the right of the peak (along the X axis) would indicate a smaller proportion of people who consume a greater amount of alcohol. Among them are the people whose levels of drinking place them at risk for different levels of alcohol problems, ranging from alcohol dependence and organ damage to early death.
2. Any two populations with the same per capita alcohol consumption will have the same prevalence of heavy drinking (e.g., more than 50 liters per year). And in any two populations with different consumption levels, the population with the higher per capita consumption will have a greater prevalence of heavy drinking. Although there seems to be a popular belief that some societies have high average alcohol consumption but low levels of alcohol abuse (France, perhaps), examples of such societies do not exist. In fact, according to Ledermann, the per capita consumption for a population group has a one-to-one relationship with the prevalence of heavy drinking.^1^

It is probably best to consider Ledermann’s two concepts as bold inferences he made from the data he gathered in his earlier epidemiological work. As it turns out, subsequent research has proven that Ledermann’s intuition in developing these concepts was sound.

## Subsequent Research

The single distribution theory achieved prominence through the work of deLint, Schmidt, and their colleagues at the Addiction Research Foundation in Toronto. Their analysis of cirrhosis mortality patterns had demonstrated a close statistical correspondence with per capita alcohol consumption, a result for which Ledermann’s theory offered a powerful explanation. During the late 1960’s, deLint and colleagues undertook a program to test the single distribution theory by examining the distribution of alcohol consumption in several data sets. They concluded that the lognormal distribution provided a reasonable approximation of the distribution of alcohol consumption (e.g., deLint and Schmidt 1968). Several Nordic researchers continued this work by analyzing the results of the distribution of alcohol consumption from general population surveys (Mäkelä 1969; Skog 1971). The two groups collaborated in writing *Alcohol Control Policies in Public Health Perspective* (Bruun et al. 1975), a project sponsored by the World Health Organization. This important monograph summarized the accumulated findings and spelled out their implications for primary prevention of alcohol-related problems.

How well have Ledermann’s two propositions held up to empirical scrutiny? Studies of a considerable number and variety of populations have accorded well with Ledermann’s first concept. The distribution of alcohol consumption does indeed have the characteristic shape of the lognormal curve—a single peak and a long tail to the right, indicating that a small proportion of the population consumes a disproportionally large amount of the total alcohol consumption for that population (Schmidt and Popham 1978; Skog 1980). This result is not surprising, because it also applies to the distributions for most consumer commodities.^2^

The second proposition is much more important and controversial, and it too has fared well over time. There is a remarkable “lawfulness” (i.e., consistency) to the distribution of alcohol consumption. For example, Skog compared several population surveys for different countries and found that the percentage of respondents reporting that they consumed more than 10 centiliters of alcohol per day “fit” Ledermann’s theory very well (Skog 1985). As predicted, this percentage was about the same for populations with similar average alcohol consumption levels, and the percentage increased with the average consumption. The latter relationship is quadratic: If population A has twice the average consumption of population B, then A has about four times the prevalence of heavy drinking.

## Significance for Policy

Ledermann’s thesis challenged the accepted wisdom of his day. At that time, most scientists and opinion leaders accepted the perspective that heavy drinking was primarily a concern because of its association with alcoholism. Alcoholics were viewed as a distinct subgroup whose drinking was largely beyond the control of alcohol availability or social context. Particularly in the United States, any proposal to raise taxes or otherwise reduce average drinking was viewed as pointless and unscientific, because these measures were based on Prohibition-era temperance thinking, the failure of which discredited such beliefs (Room 1984).

In contrast, Ledermann took the epidemiologist’s view, that whether or not heavy drinkers were in some sense alcoholics, they were at risk for a variety of life-threatening illnesses. Ledermann also asserted that drinking, even heavy drinking, was influenced by the drinker’s social environment. Indeed, an implication of the single distribution theory is that any intervention reducing per capita consumption necessarily reduces the prevalence of heavy drinking.^3^ Furthermore, this theory suggests the futility^4^ of attempting to reduce the prevalence of heavy drinking without reducing normal consumption levels. Thus the primary prevention of health problems stemming from chronic heavy drinking becomes closely linked with reducing overall consumption within the whole population. Such measures as raising taxes on alcoholic beverages, reducing density of outlets that sell alcoholic beverages, and restricting alcoholic beverage advertising become, in this perspective, the key weapons in combatting the diseases resulting from chronic heavy drinking (Edwards et al. 1994).

Ledermann’s intuition concerning the distribution of alcohol consumption captured important elements of this phenomenon. Heavy drinkers, whether or not they are alcoholics, are not a distinct group immune to social and economic pressure but rather are part of a continuum with moderate and light drinkers. There is both good news and bad news here for alcohol control policy. Ledermann’s work shows that the heavy drinkers will reduce their drinking if—but only if—the others cut back too.

## Footnotes

1 Ledermann proposed a formula relating the mean of the (lognormal) drinking distribution to its standard deviation. Given this formula, knowledge of a population’s average consumption was sufficient to characterize completely the drinking distribution.

2 It should be noted that the lognormal distribution is by no means the only one with this shape, and in some studies, it appears that one of these other distributions provides a somewhat better “fit” to the data than does the lognormal.

3 See Pitman (1980) for a critique of this conclusion.

4 One exception has been documented. The Swedish Bratt System for rationing sales of alcoholic beverages was apparently successful at eliminating much of the upper tail of the alcohol consumption distribution (Norström 1987).
